# Clinical amyloid and tau positron emission tomography imaging in Alzheimer's disease: image interpretation in the era of anti-amyloid therapies

**DOI:** 10.1097/WCO.0000000000001471

**Published:** 2026-02-19

**Authors:** Gregory Mathoux, Elif Harput, Débora E. Peretti, Cecilia Boccalini, Valentina Garibotto

**Affiliations:** a Division of Nuclear Medicine and Molecular Imaging, Geneva University Hospitals; b Laboratory of Neuroimaging and Innovative Molecular Tracers (NIMTLab); c Centre for Biomedical Imaging, University of Geneva, Geneva, Switzerland

**Keywords:** Alzheimer's disease, amyloid, anti-amyloid treatment, positron emission tomography, tau

## Abstract

**Purpose of review:**

The introduction of disease-modifying anti-amyloid therapies has shifted the role of positron emission tomography (PET) imaging in Alzheimer's disease from confirming diagnosis to actively guiding clinical decision-making within the AT(N) framework.

**Recent findings:**

Amyloid PET has become central for confirming treatment eligibility, quantifying biological response, and supporting PET-guided strategies for treatment duration, particularly through standardized visual interpretation and Centiloid-based quantification. Tau PET provides complementary information by reflecting disease stage and the burden of pathology most closely associated with cognitive impairment, thereby helping to contextualize expected clinical benefit. Recent clinical trials have integrated PET imaging to monitor therapeutic effects and to support translation into routine clinical practice.

**Summary:**

This review focuses on practical aspects of visual interpretation and semi-quantitative analysis of amyloid and tau PET, discusses tracer-specific considerations and ongoing harmonization efforts, and summarizes the expanding clinical role of PET imaging. Together, amyloid and tau PET support a more biologically grounded and individualized approach to Alzheimer's disease care in the era of disease-modifying therapies.

## INTRODUCTION

Alzheimer's disease (AD) is now increasingly viewed as a biologically defined continuum rather than a purely clinical syndrome, with biomarkers of amyloid deposition, tau pathology, and neurodegeneration providing a structured framework for in vivo disease characterization and staging [1^▪^]. This review focuses on the visual interpretation and semi-quantitative use of amyloid and tau positron emission tomography (PET) within this framework, while acknowledging that additional molecular imaging targets reflecting neurodegeneration, neuroinflammation, synaptic dysfunction, and co-pathologies are critical for capturing disease heterogeneity and are discussed in detail elsewhere [2]. Moreover, we discuss direct implications of this imaging-based biological framework for patient selection, prognostication, and treatment guidance in the current era of disease-modifying therapies. 

**Box 1:**
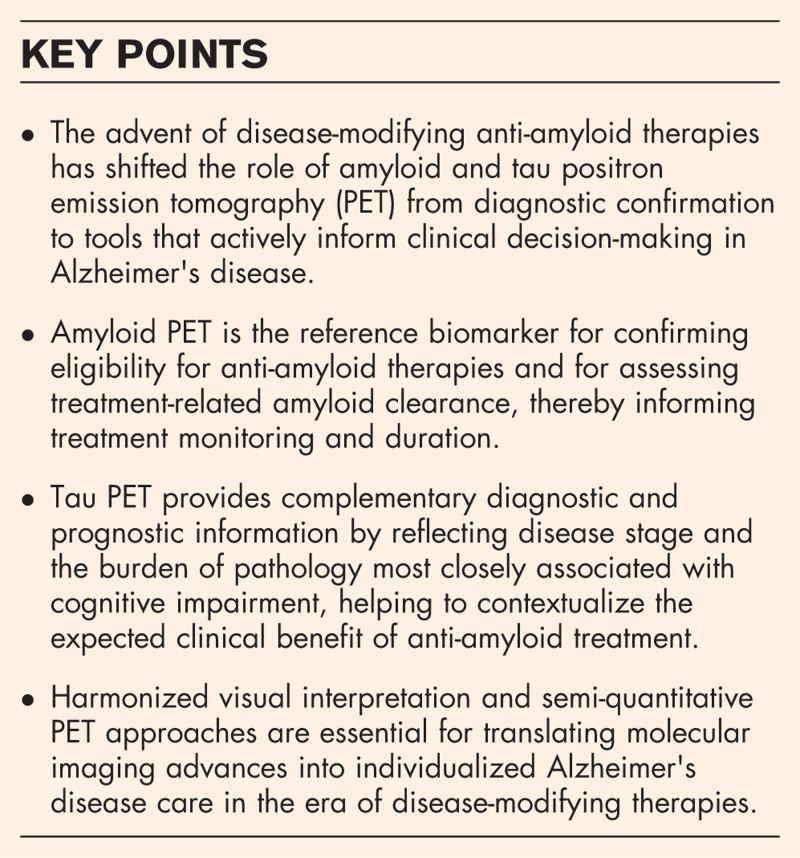
no caption available

## AMYLOID POSITRON EMISSION TOMOGRAPHY: VISUAL INTERPRETATION AND SEMI-QUANTIFICATION STRATEGIES

Amyloid-beta deposition constitutes the “A” component of the AT(N) classification framework and can be reliably detected in vivo using amyloid PET imaging. Extensive validation studies have demonstrated strong correlations between amyloid PET tracer uptake, postmortem amyloid burden, and established neuropathological staging systems, supporting amyloid PET as a robust biomarker and a cornerstone of the biological definition of AD [3].

The first amyloid-PET tracer to demonstrate feasibility in humans was [^11^C]Pittsburgh compound B ([^11^C]PiB), which exhibits high affinity for fibrillar amyloid-beta plaques. However, its short half-life limits routine clinical use, motivating the development of fluorine-18–labeled tracers suitable for widespread implementation [4]. Currently approved tracers, including [^18^F]Florbetapir, [^18^F]Florbetaben, and [^18^F]Flutemetamol, have enabled broad clinical adoption of amyloid PET [5]. While these agents target the same pathological substrate, they differ in pharmacokinetics, image contrast, and degrees of nonspecific white matter binding. These differences necessitate tracer-specific interpretation criteria and careful reader training. Nonetheless, head-to-head studies consistently demonstrate high concordance among tracers for binary amyloid classification, supporting their interchangeable use in clinical practice [3].

**FIGURE 1:**
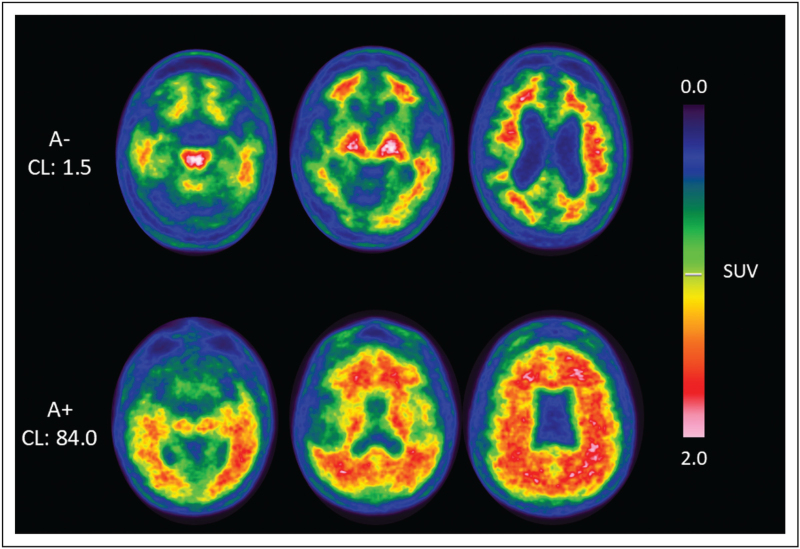
The figure shows representative amyloid PET images acquired with [^18^F]-Flutemetamol. The first row illustrates a scan classified as negative on visual assessment, characterized by preserved gray–white matter differentiation and absence of significant cortical tracer binding, with concordant semi-quantitative analysis yielding a Centiloid value of 1.5. The second row illustrates a scan with diffuse cortical tracer uptake, visually classified as positive and confirmed by semi-quantitative analysis with a Centiloid value of 84.0.

Visual assessment of amyloid-PET images relies on a standardized binary classification framework. A scan is considered amyloid-positive when there is reduced gray–white matter contrast as well as when the radiotracer uptake extends to the cortical surface and forms continuous pattern along the cerebral cortex. In contrast, amyloid-negative scans are characterized by preserved gray–white matter difference and the absence of significant cortical tracer binding. Operational criteria vary depending on the radiotracer used [6,7]. For [^18^F]Florbetapir, positivity is defined by cortical uptake exceeding white matter uptake in two or more regions. For [^18^F]Florbetaben and [^18^F]Flutemetamol, increased uptake in at least one major cortical region (frontal, lateral temporal, parietal, or posterior cingulate/precuneus) is sufficient, typically using cerebellar cortex as the reference region (Fig. 1). While visual interpretation remains the clinical standard, semi-quantitative approaches provide complementary information. The standardized uptake value ratio (SUVR), calculated relative to a reference region such as whole cerebellum or pons, improves reproducibility, supports longitudinal monitoring, and increases confidence in visually borderline cases. However, SUVR values are influenced by the tracer chemistry, target and reference region choice and processing parameters, limiting direct comparability across tracers and centers.

The Centiloid framework was developed to address this limitation by harmonizing amyloid PET measurements on a common scale, anchored at 0 for young controls and 100 for typical AD-level amyloid burden [8]. Application of this common quantitative scale facilitates consistent diagnosis, prognosis, and monitoring of disease progression, as well as comparison across studies and therapeutic trials. High inter-reader agreement has been demonstrated for visual reads by experienced interpreters, with strong concordance between visual classification and Centiloid-based quantification [9,10]. In visually challenging or borderline cases, semi-quantitative measures can further enhance diagnostic confidence and reduce uncertainty [11^▪^]. Alongside this framework, simplified quantitative approaches are being developed to facilitate wider clinical use, including PET-only methods that do not require concomitant MRI and are implemented in CE-approved software solutions, which have shown good agreement across different quantification methods [12,13]. In an internal validation on a subset of 10, PET-only quantification implemented in the certified syngo.via platform (Siemens Healthineers) was compared with an established MRI-based research pipeline following the Klunk method, showing very high agreement (*r* ≈ 0.98), supporting their validity for routine clinical use.

## TAU POSITRON EMISSION TOMOGRAPHY: VISUAL INTERPRETATION AND SEMI-QUANTIFICATION STRATEGIES

Tau pathology in AD is defined by the accumulation of neurofibrillary tangles composed of paired helical filaments, typically following a regional progression described by Braak staging [14]. Tau PET imaging enables in vivo visualization of both the spatial distribution and burden of tau pathology, providing information that complements amyloid PET and more closely reflects disease stage and clinical severity.

First-generation tau PET tracers, including [^18^F]Flortaucipir, [^11^C]PBB3, and the [^18^F]THK family, have been extensively used in research but are limited by off-target binding, particularly in the choroid plexus, basal ganglia, meninges, and certain midbrain structures. Such nonspecific uptake can complicate visual interpretation and quantification, especially in adjacent regions such as the hippocampus. Partial volume correction and subject-space processing has been proposed to mitigate spill-in effects, but its application remains inconsistent, and prior studies suggest limited incremental benefit in routine clinical interpretation [15,16]. To address these limitations, second-generation tau-PET tracers have been developed with improved binding specificity and reduced nonspecific uptake. These include [^18^F]MK6240, [^18^F]RO948, [^18^F]PI2620, [^18^F]GTP1, and [^18^F]JNJ-64326067, all of which demonstrate more favorable kinetic and binding profiles compared with earlier tracers [16]. Despite these advances, [^18^F]Flortaucipir remains the only tau tracer approved for clinical use, with regulatory approval in both the United States and Europe.

**FIGURE 2:**
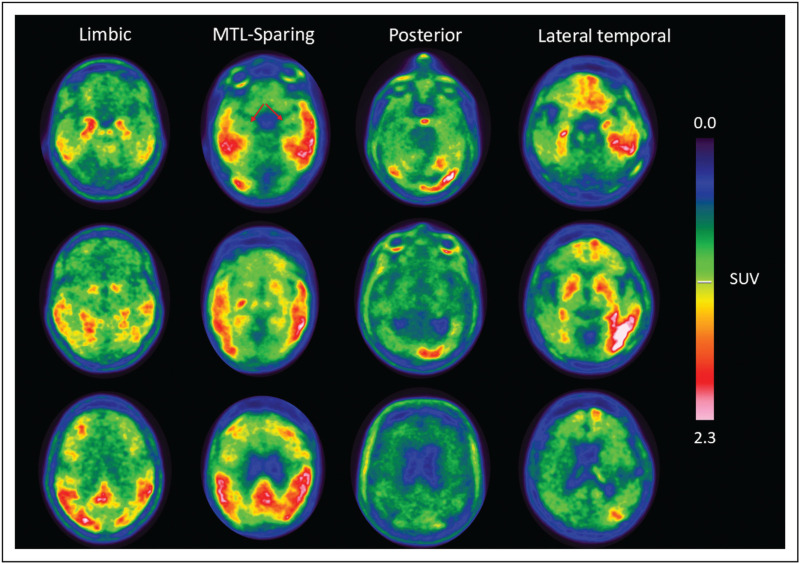
Four distinct individual [18F]-Flortaucipir PET spatial patterns that can be visually identified, as described in previous literature [19^▪^]. The first column shows an example of the *limbic* phenotype, characterized by a typical Braak-like distribution. The second column shows the *medial temporal lobe–sparing* phenotype, with prominent temporoparietal cortical uptake and relative sparing of the medial temporal lobes as showed by the red arrows. The third column illustrates the *posterior* phenotype, with predominant occipitotemporal accumulation. The fourth column shows the *lateral temporal* phenotype, characterized by a clearly predominant left-sided temporoparietal accumulation.

Visual interpretation of tau PET in clinical practice is based on a binary framework anchored in Braak staging. Scans are considered positive for AD when elevated tracer uptake is observed in posterolateral temporal, parietal, and occipital cortices, corresponding to Braak stages IV–VI. Uptake confined to medial temporal regions, consistent with Braak stages I–III, is generally considered negative in the context of AD diagnosis [17]. Nevertheless, tau PET distribution shows a relevant inter-individual variability of patterns along the described progression stages and at least four spreading patterns have been identified and are visually detectable [18,19^▪^], as exemplified in Fig. 2.

When performing visual reads, careful consideration of the color scale is essential, as most proposed interpretation methods recommend the use of high-contrast color scale to optimize detection of pathological uptake. Appropriate selection of color scales is critical, as high-contrast maps improve detection of pathological uptake and reduce reader variability. Visual tau PET interpretation has demonstrated robust associations with cognitive impairment and disease stage, supporting its clinical relevance [20]. In addition to visual assessment, semi-quantitative methods are commonly used in both research and clinical contexts to complement visual reads. Reference region selection is a key methodological consideration, with cerebellar gray matter, inferior cerebellum, or cerebellar crus commonly used to minimize spill-in and off-target effects [16]. Meta-regions of interest (meta-ROIs) are also widely applied in semi-quantitative analyses. These composite regions were developed to account for inter-individual variability in tau deposition, including cases that show neocortical tau accumulation beyond the classical Braak framework. Accordingly, meta-ROIs typically include regions reflecting both early and late stages of tau pathology in AD [21].

Emerging harmonization frameworks, such as CenTauR and UniT, aim to standardize tau PET measurements across tracers and centers, miming the Centiloid scale per amyloid-PET, although further validation is required [22,23]. Although visual assessment remains the primary method used in clinical practice, semi-quantitative approaches play an important complementary role. Previous studies have demonstrated good agreement between visual classifications and SUVR-based thresholds, supporting the combined use of both methods to enhance diagnostic confidence [20,24].

## POSITRON EMISSION TOMOGRAPHYIMAGING IN ANTI-AMYLOID CLINICAL TRIALS

In anti-amyloid clinical trials, PET imaging has evolved into a core methodological pillar, extending beyond its role as a diagnostic screening tool. Amyloid PET is used both to confirm biological eligibility – ensuring sufficient baseline amyloid burden – and to quantify therapeutic efficacy by measuring plaque removal longitudinally. The adoption of the Centiloid scale was instrumental in this evolution, providing a standardized quantitative framework that enabled comparison across tracers, scanners, and study sites and allowing treatment effects to be interpreted on an absolute biological scale.

Amyloid PET has been a mandatory inclusion criterion in most recent phase II and III trials of anti-amyloid monoclonal antibodies. In the phase 3 Aducanumab trial (EMERGE and ENGAGE), amyloid-PET was part of eligibility criteria, and PET studies demonstrated dose- and time-dependent reductions in amyloid burden [25]. The CLARITY-AD trial of Lecanemab showed rapid, substantial, and sustained amyloid reductions with clear separation from placebo within months of treatment initiation. At 18 months, mean amyloid reduction approached 55 Centiloids in the active treatment group and was accompanied by statistically significant slowing of clinical decline [26].

In the phase 2 TRAILBLAZER-ALZ trial, donanemab induced very large reductions in amyloid plaque burden, exceeding 80 Centiloids at 76 weeks compared with placebo [27]. In phase 3 TRAILBLAZER-ALZ 2 trial, again an important reduction of amyloid burden was found, with more than three quarters of treated participants achieving amyloid-negative status. Crucially, treatment was discontinued in a blinded manner once predefined low amyloid thresholds were reached (11 Centiloids on a single scan or if two consecutive scans demonstrated values between 11 and 25 Centiloids), transforming amyloid PET from a passive outcome measure into an active decision-making tool [28]. Post hoc analyses demonstrated durable amyloid clearance, with minimal reaccumulation over one year and a prolonged estimated time (approximately 3.9 years) to return to PET positivity [29].

Across trials, PET imaging revealed marked interindividual variability in biological response. Importantly, secondary analyses consistently showed that lower posttreatment amyloid levels – not simply change from baseline – were strongly associated with slower cognitive and functional decline [30^▪^]. These findings suggest that the timing and completeness of amyloid clearance are critical determinants of clinical benefit. Near-complete amyloid removal within approximately 18 months appears to maximize treatment effect, supporting PET-guided strategies to individualize treatment duration, reduce cumulative exposure, and potentially mitigate adverse effects [31].

Tau PET was incorporated into selected trials as a complementary biomarker to contextualize amyloid removal and biological heterogeneity rather than as a primary efficacy endpoint. In TRAILBLAZER-ALZ, tau PET was used at screening to restrict enrollment to participants with low or intermediate tau burden, and longitudinal analyses suggested relative slowing of regional tau accumulation in participants achieving substantial amyloid clearance [27–29]. In CLARITY-AD, post hoc analyses similarly indicated greater clinical benefit in individuals with low, or absent, baseline tau burden, reinforcing tau PET as a marker of disease stage and treatment responsiveness rather than a direct efficacy endpoint [32].

## CLINICAL USE OF AMYLOID AND TAU PET IMAGING FOR ANTI-AMYLOID TRETAMENT: PATIENT SELECTION AND TREATMENT MONITORING

With the FDA and EMA recent approval of Lecanemab and Donanemab for clinical use, amyloid PET has become a routinely used biomarker for the clinical implementation of anti-amyloid therapy. Both agents are approved for patients with early symptomatic Alzheimer's disease and mandate confirmation of cerebral amyloid pathology prior to treatment initiation. Appropriate use recommendations emphasize amyloid PET as a reliable and standardized method for establishing the presence of the therapeutic target, particularly in patients with diagnostic uncertainty or atypical clinical presentations [33,34]. In real-world practice, amyloid PET has become the preferred modality for biomarker confirmation in many centers, given its direct visualization of the therapeutic target, established pathological validity, and quantitative harmonization through the Centiloid scale. Beyond a binary positive–negative classification, quantitative interpretation of amyloid PET using the Centiloid scale is increasingly advocated to support consistent clinical decision-making. Expert consensus proposes pragmatic eligibility thresholds in the range of approximately 24–30 Centiloids, which balance diagnostic certainty with avoidance of unnecessary treatment in individuals with minimal amyloid burden [35]. This quantitative framework is particularly important as anti-amyloid therapies move beyond specialized trial environments into routine clinical settings with heterogeneous expertise and infrastructure. Beyond patient selection, amyloid PET is increasingly informing treatment monitoring and duration, especially for donanemab. As highlighted by Rabinovici *et al.*, donanemab is the first anti-amyloid therapy to operationalize PET-guided treatment discontinuation, a strategy already embedded in phase 3 trial protocols and directly translatable to clinical care [34]. In TRAILBLAZER-ALZ 2, treatment was discontinued once low amyloid thresholds are reached with lower posttreatment amyloid levels associated with slower cognitive and functional decline and amyloid clearance appears durable when very low Centiloid values are achieved. In parallel, a recently published expert workgroup has proposed defining treatment-related amyloid clearance (TRAC) using amyloid PET, emphasizing the role of quantitative measurements in characterizing the degree of clearance achieved [36^▪▪^]. his strategy has important implications for reducing patient burden, infusion center demands, cumulative costs, and long-term exposure to treatment-related risks [30^▪^,^34^,^37^^▪▪^].

Tau PET currently plays a complementary role in this evolving clinical paradigm. Although not required for treatment initiation or routine monitoring, tau PET provides critical biological context by reflecting disease stage. Baseline tau burden influences the likelihood that amyloid removal will translate into meaningful clinical benefit, positioning tau PET as a prognostic rather than therapeutic biomarker [38]. In clinical practice, tau PET may inform patient counseling and risk–benefit discussions, particularly in borderline cases or in patients at higher risk for treatment-related complications such as amyloid-related imaging abnormalities (ARIA). In such scenarios, high baseline tau burden may argue against treatment due to limited anticipated benefit, whereas low tau burden may favor therapy by shifting the risk–benefit balance toward greater expected clinical response. While broader implementation remains limited by availability and standardization, tau PET is poised to play an increasingly important role in precision-guided use of anti-amyloid therapies [37^▪▪^].

## CONCLUSION

The advent of disease-modifying anti-amyloid therapies has reshaped the clinical role of molecular neuroimaging in AD, positioning amyloid and tau PET as integral component of patient selection, and therapeutic decision-making rather than purely diagnostic biomarkers. Amyloid PET is now central for confirming treatment eligibility, quantifying biological response, and enabling PET-guided strategies that individualize treatment duration in routine clinical practice. Tau PET, by reflecting disease stage and the burden of pathology most closely associated with cognitive impairment, provides essential prognostic context and helps frame realistic expectations regarding clinical benefit. As standardization improves and tau-directed therapies trials advance, the clinical relevance of tau PET is likely to expand further, reinforcing the central role of PET imaging in precision-guided care for AD.

## Acknowledgements

*V.G. received research support and speaker fees through her institution from GE Healthcare, Siemens Healthineers, Novartis, Eli Lilly and Roche.*

### Financial support and sponsorship

*None.*

### Conflicts of interest

*None.*

## REFERENCES AND RECOMMENDED READING

Papers of particular interest, published within the annual period of review, have been highlighted as:

▪ of special interest
▪▪ of outstanding interest
